# Liquid Phase Sintering of Al Powder Using Al-X (X=Cu, Ca, Mg) Eutectic Alloy Powders: Effect of Alloy Elements and Oxide Film Thickness

**DOI:** 10.3390/ma18081755

**Published:** 2025-04-11

**Authors:** Ryotaro Kusunoki, Hideaki Hayashi, Erika Matsumoto, Asuka Suzuki, Naoki Takata, Makoto Kobashi, Akira Yoshida, Takahiro Hamada, Moe Mekata

**Affiliations:** 1 Department of Materials Process Engineering, Graduate School of Engineering, Nagoya University, Furo-cho, Chikusa-ku, Nagoya 464-8603, Japan; kusunoki.ryotaro.r1@s.mail.nagoya-u.ac.jp (R.K.); hayashi.hideaki2357@gmail.com (H.H.); matsumoto.erika.j0@s.mail.nagoya-u.ac.jp (E.M.); kobashi.makoto.s2@f.mail.nagoya-u.ac.jp (M.K.); 2 Department of Materials Design Innovation Engineering, Graduate School of Engineering, Nagoya University, Furo-cho, Chikusa-ku, Nagoya 464-8603, Japan; takata.naoki@material.nagoya-u.ac.jp; 3 Nissan Motor Co., Ltd., 2, 1, Natsushima-cho, Yokosuka-shi 237-8523, Japan; a-yoshida@mail.nissan.co.jp (A.Y.); t-hamada@mail.nissan.co.jp (T.H.); moe-mekata@mail.nissan.co.jp (M.M.)

**Keywords:** pressure-less liquid phase sintering, aluminum powder, eutectic alloy, oxide film, additive manufacturing

## Abstract

Sinter-based additive manufacturing (AM) requires sintering for the densification of green bodies. Al powder is difficult to sinter due to the dense oxide film on the surface, and it is difficult to apply to sinter-based AM. Liquid phase sintering using Al-based eutectic alloy powder is promising for sintering Al powder without external pressure. In this study, Al powders with various oxide film thicknesses were sintered using Al-X eutectic alloy powders (X=Cu, Ca, and Mg) to clarify suitable alloy elements in the sintering aids for the liquid phase sintering. When an as-supplied Al powder with an oxide film thickness of approximately 2 nm (presumably amorphous Al_2_O_3_ film) was used, Al-Cu and Al-Ca aids promoted the densification, whereas numerous pores were observed in the sample sintered using Al-Mg aid. The pores would be formed during the cooling after sintering, along with the homogenization of Mg distribution. When Al powder with an oxide film thickness of around 4 nm was used, a high relative density of over 95% was maintained using Al-Cu aid, whereas the relative density of the sample sintered using Al-Ca aid significantly degraded, presumably due to the formation of Ca-based oxide. These results indicate that the Al-Cu eutectic alloy powder is a promising sintering aid for the liquid phase sintering of Al powder.

## 1. Introduction

Recently, metal additive manufacturing (AM) has been attracting attention as a process that can produce metal parts with complex shapes [1]. Metal AM processes such as powder bed fusion (PBF) [2] and directed energy deposition (DED) [3] have been widely studied. These processes involve the melting of raw material powders or wires to create three-dimensional objects. Processes such as binder jetting (BJT) [4] and material extrusion (MEX) [5] have also been studied. In BJT, metal powder is bedded, and an organic binder is injected into the powder bed to bind the metal particles. Three-dimensional green bodies are produced by repeatedly bedding metal powder and selectively injecting binder. In MEX, filaments consisting of a mixture of metal powder and resin are extruded through a nozzle and stacked in layers to create a three-dimensional green body. Both BJT and MEX produce dense products through the process of debinding and sintering.

Major materials under study include titanium alloys [6], nickel-based superalloys [7], stainless steels [8], and copper alloys [9]. Aluminum (Al) and its alloys have also been applied to these processes. However, there are still challenges in applying aluminum and its alloy powders to these processes. The oxide film covering the surface of Al powder inhibits sintering, making it difficult to manufacture dense Al products [10,11]. Recently, supersolidus liquid phase sintering using pre-alloyed Al powders has been applied as a sintering process of BJT [12,13,14,15]. The supersolidus liquid phase sintering of pre-alloyed Al alloy powder has been investigated in Al-Si [15], Al-Si-Mg [13,14], and Al-Cu-Mg [16] alloy systems. For example, AlSi10Mg pre-alloyed powder commonly used in PBF and DED was processed by BJT and sintered at a temperature higher than the solidus temperature. Then, the pre-alloyed powder was partially melted, and the liquid phase promoted the densification of the green body [13,14]. Furthermore, the Al-Si alloy heat sinks were fabricated by BJT and a subsequent supersolidus liquid phase sintering process [14]. However, it is necessary to develop and understand the sintering processes of Al powders further to extend the options of Al alloys that can be applied to BJT and MEX.

The sintering of Al powders is usually carried out under pressure [16]. The oxide film on the particle surface is crushed by plastic deformation of the powder by applying pressure at high temperatures, promoting the densification. However, external pressure is difficult to apply to complex-shaped green bodies that are fabricated by BJT or MEX.

As another technique, a liquid phase sintering process using elemental powders has been proposed. In this process, elemental powders with a lower melting point than Al (Sn, Zn, etc.) or with a eutectic-type phase diagram with Al (Cu, Si, Mg, etc.) are blended with Al powder and sintered [17,18]. When an element with a lower melting point than Al is added, the elementary powder generates a liquid phase. When elemental powders with a eutectic phase diagram are added to Al, interdiffusion between Al and the added element powders locally lowers the melting temperature and generates a liquid phase, promoting the densification of the green body. In this process, no external force needs to be applied during sintering. Elements with melting points lower than that of Al are limited to those that are not generally used for Al alloys, except for Zn. Zn is not preferable for promoting the sintering of Al powder because the solid solubility limit of Zn in Al is high (maximum solid solubility: 67% [19]). The large solid solubility in Al causes swelling rather than densification [20]. In contrast, elements with eutectic-type phase diagrams with Al include those that are commonly used for Al alloys and are suitable for strengthening the Al alloy sintered products after sintering. It is also reported that the oxide film can be reduced by appropriately selecting the additive elements. A typical example is Mg, which causes the following reaction with alumina [21].

3Mg + 4Al_2_O_3_ ↔ 3MgAl_2_O_4_ + 2Al

Here, the MgAl_2_O_4_ has a spinel-type crystal structure. It is believed that the change in volume due to the formation of MgAl_2_O_4_ causes shear stress in the oxide film, leading to its fracture [22]. In the liquid phase sintering using elemental powders, generating a eutectic reaction with Al, the relative density of the sintered body strongly depends on the relative density of the green body [23]. To increase the relative density of the sintered body, the relative density of the green body must also be increased to increase the contact area between Al and added powders for efficient interdiffusion. However, no external force is applied during the fabrication of the green body through BJT, resulting in a relatively low relative density of the green body (40–60%) [24]. The volume fraction of metal powders in the filament of MEX (defined as solid loading) is limited to 50–70% to smoothly extrude the filament from the nozzle [25]. Thus, the relative density of the green body in BJT and MEX cannot always be increased. That is why the liquid phase sintering using elemental powders that cause a eutectic reaction with Al is unsuitable for BJT and MEX.

As another approach, a liquid phase sintering process using Al-Si eutectic alloy powder as a sintering aid with a low melting temperature has recently been reported [26]. In this process, Al powder is blended with Al-Si eutectic alloy powder and sintered at a temperature higher than the eutectic temperature. The Al-Si eutectic alloy powder is fully melted, and the liquid phase promotes densification. A high relative density of approximately 95% is achieved from a green body with a relative density of approximately 75%. This process is also promising as a sintering process for green bodies fabricated by BJT and MEX because it can densify green bodies with relatively low relative density. Since Al has eutectic phase diagrams with many elements, there are many alloy elements that can be used as sintering aids. In addition, many elements exhibiting eutectic-type phase diagrams with Al are often used for strengthening Al alloys (e.g., Cu, Si, Mg, and Zn). Therefore, elements contained in sintering aids that promote sintering can be utilized to strengthen the sintered Al powder compact through the solution and aging heat treatments after sintering. However, it is not known which eutectic alloy powders are suitable as liquid phase sintering aids for Al powder.

The oxidation state of Al powder will significantly affect its densification during liquid phase sintering. Al powder is manufactured through various processes, including Ar or N_2_ gas atomization, water atomization, air atomization, mechanical crushing, and thermal plasma. The oxidation state as well as the morphology of Al powders will depend on the process (e.g., air-atomized powder is more oxidized than Ar or N_2_ gas-atomized powder). It is preferable that the liquid phase sintering aids for Al powder be able to promote its densification regardless of its oxidation state. However, the impact of the oxidation state on the liquid phase sintering behaviors of Al powder has not been investigated. As mentioned above, some elements, including Mg and Ca, react with alumina [21,27]. If these elements in Al-based eutectic alloy powders as sintering aids react with alumina, there is a possibility that the sintering aids can promote the densification of Al powder regardless of its oxidation state. Thus, the sintering behaviors of Al powders with different oxidation states using various Al-based eutectic alloy powders (containing Mg, Ca, and others) need to be clarified.

In this study, the liquid phase sintering of Al powder was performed using Al-X eutectic alloy powders (X = Cu, Mg, and Ca) as sintering aids. The relative density and microstructure of the sintered compacts were investigated. In addition to high-purity Al powder with a relatively thin oxide film, the liquid phase sintering of Al powder with an oxide film grown by oxidation heat treatment in air was also carried out using the same aids, and the relative density and microstructure were investigated. Based on these results, the screening of suitable elements for eutectic alloy powders as sintering aids for Al powder with different oxidation states was conducted.

## 2. Experimental Procedure

### 2.1. Selecting Elements in Eutectic Alloy Powder, Sintering Temperature, and Blending Compositions

Table 1 shows the characteristics of the sintering aids used in this study, based on the equilibrium phase diagrams for Al-Mg, Al-Ca, and Al-Cu binary systems [28]. The Al-Mg binary system has a eutectic composition of Al-37 mol%Mg (α/Al_45_Mg_28_ eutectic) and a eutectic temperature of approximately 450 °C [29]. In addition, as mentioned above, Mg will promote sintering by reducing alumina. The Al-37 mol%Mg eutectic alloy powder (hereinafter referred to as “Al-Mg aid”) was selected as a sintering aid in this study. The Al-Ca binary system has a eutectic composition of Al-5.5 mol%Ca (α/Al_4_Ca eutectic) and a eutectic temperature of approximately 613 °C [30]. It has been reported that Ca also reacts with alumina to form CaAl_2_O_4_ [27]. Therefore, this study also selected Al-5.5 mol%Ca eutectic alloy powder (hereinafter referred to as “Al-Ca aid”) as a sintering aid. Based on the Ellingham diagram [31], Mg and Ca have higher negative standard free energies of oxide formation than Al, indicating that Mg and Ca have a higher affinity for oxygen than Al. As a result, these elements react with alumina. In contrast, Cu has a much lower standard free energy of oxide formation than Al [32] and does not react with alumina. The Al-Cu binary system has a eutectic composition of Al-17.3 mol% (α/Al_2_Cu eutectic) and a eutectic temperature of 548 °C [28]. Therefore, Al-17.3 mol%Cu eutectic alloy powder (hereafter referred to as “Al-Cu aid”) was also selected as a sintering aid.

**Figure 1:**
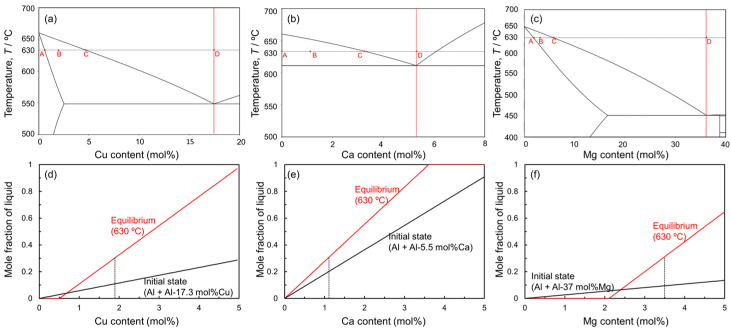
Equilibrium phase diagrams for (**a**) Al-Cu, (**b**) Al-Ca, and (**c**) Al-Mg binary systems. The compositions of A, B, C, and D in the phase diagrams indicate the solid solubility limit at 630 °C, the studied composition, the equilibrium composition of the liquid phase at 630 °C, and the eutectic composition. Changes in mole fraction of liquid phase at initial and equilibrium states at 630 °C as a function of (**d**) Cu, (**e**) Ca, and (**f**) Mg contents.

Figure 1 shows the equilibrium phase diagrams of (a) Al-Cu, (b) Al-Ca, and (c) Al-Mg binary systems. In this liquid phase sintering process, Al powder is mixed with Al-Cu, Al-Ca, and Al-Mg aids. The sintering temperature must be below the melting point of the Al powder (660 °C) and above the eutectic temperatures of the eutectic alloy powders. The highest eutectic temperature among the aids used in this study is 613 °C for the Al-Ca aid. Therefore, 630 °C was selected as the sintering temperature in this study. In liquid phase sintering, the fraction of the liquid phase during sintering is one of the important factors. The liquid-phase fraction in the liquid phase sintering is usually in the range of 10–30% [20]. In this liquid phase sintering process, two types of liquid phase fractions can be defined. One is the initial liquid fraction, which is defined only by the mixing ratio of Al powder and each aid. Assuming that only the aids melt completely when the sample temperature exceeds the eutectic temperature, the mixing ratio of the aids determines the initial liquid phase fraction. The other is the equilibrium liquid phase fraction. This fraction is determined by the sintering temperature and mixing ratio and is obtained by the lever rule in the solid–liquid two-phase region of the equilibrium phase diagram. Based on the above, changes in the initial liquid phase fraction above the eutectic temperatures and the equilibrium liquid phase fraction at 630 °C as a function of the concentration of each alloy element are shown in Figure 1d,e. In this study, Al and each aid are mixed at a composition that yields an equilibrium liquid phase fraction of 30 mol%, considering that atomic diffusion in the liquid phase is rapid and the liquid phase fraction presumably reaches the equilibrium state at the early stage of sintering. Specifically, Al powder was mixed with Al-Cu aid to make an average composition of Al-1.9 mol%Cu, with Al-Ca aid to make an average composition of Al-1.1 mol%Ca, and with Al-Mg aid to make an average composition of Al-3.5 mol%. It is generally known that densification occurs when the dissolution of atoms from the solid phase to the liquid phase is dominant in the early sintering stage, while swelling occurs when the dissolution of atoms from the liquid phase to the solid phase is dominant [33]. As shown in Figure 1d−f, the equilibrium liquid phase fraction increases relative to the initial liquid phase fraction under the conditions selected in this study. This indicates that the dissolution of Al atoms from the Al powder into the liquid phase is more pronounced than the dissolution of alloy element atoms from the liquid phase into the Al powder. Thus, it is expected that densification will occur under the selected conditions.

### 2.2. Sample Preparation and Evaluation

**Figure 2:**
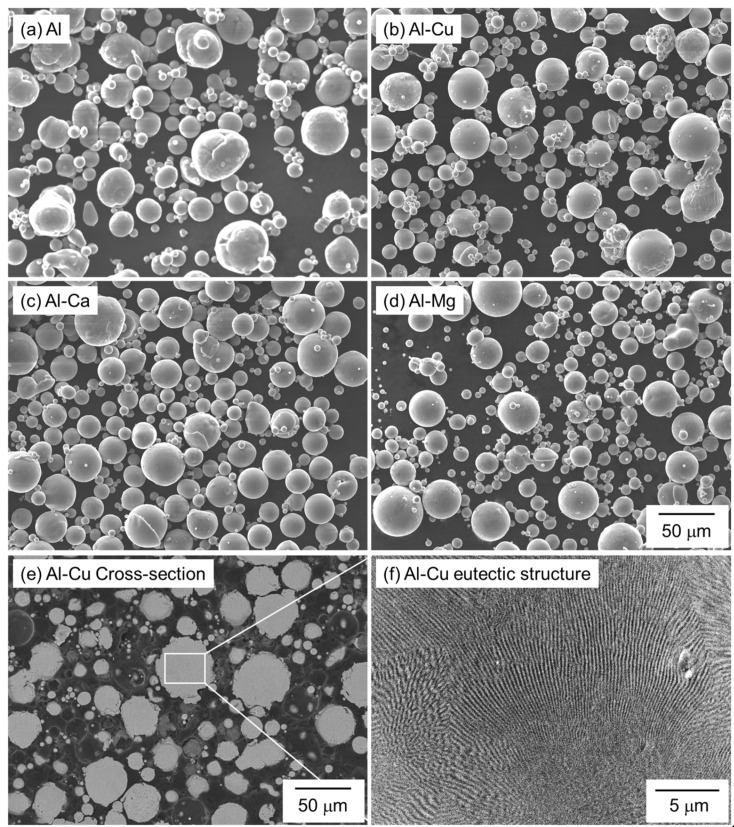
SEM images showing the morphology of (**a**) pure Al, (**b**) Al-Cu, (**c**) Al-Ca, and (**d**) Al-Mg powders. (**e**) Low- and (**f**) high-magnification cross-sectional SEM images showing the microstructure of Al-Cu powder.

Spherical Al (particle size: <45 μm, purity 99.99%, KOJUNDO CHEMICAL LABORATORY Co., Ltd., Saitama, Japan), Al-Cu, Al-Mg, and Al-Ca powders (particle size: <45 μm, KOJUNDO CHEMICAL LABORATORY Co., Ltd., Saitama, Japan) produced by Ar gas atomization were used as raw materials. Figure 2 shows the SEM images showing the morphology of the powders. Table 2 shows the compositions of the Al-Cu, Al-Mg, and Al-Ca aids measured by inductively coupled plasma and infrared absorption methods. The Cu, Mg, and Ca compositions of all the aids are close to the target eutectic compositions. Figure 2e,f shows the cross-sectional microstructures of the Al-Cu aid. Since the Al-Cu aid is fabricated by gas atomization, which has a relatively high cooling rate, a fine eutectic structure can be observed inside the Al-Cu aid. The Al-Cu aid used in this study is a eutectic alloy powder with a low melting temperature.

**Figure 3:**
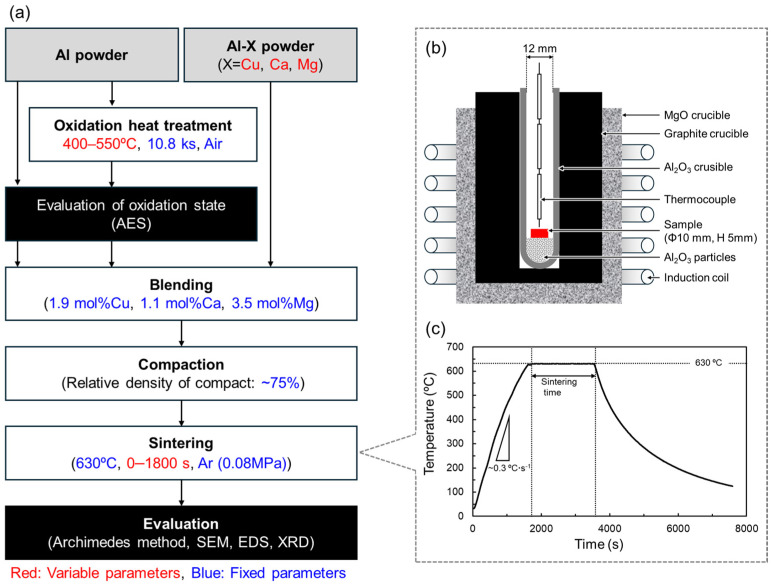
(**a**) Flow chart of experimental procedure in this study. The white, grey, and black rectangles indicate processes, materials, and evaluations, respectively. The variable and fixed process parameters are colored red and blue, respectively. (**b**) Schematic illustration showing the setup for sintering using an induction heating furnace. (**c**) Temperature history during the liquid phase sintering of Al powder.

The overall experimental flow and associated process parameters are summarized in Figure 3. The white, grey, and black rectangles indicate processes, materials, and evaluations, respectively. The variable and fixed process parameters are colored red and blue, respectively.

To change the oxidation state of the Al powder surface, oxidation heat treatments were performed in an electric furnace (FO101, Yamato Scientific Co., Ltd. Tokyo, Japan). In general, alumina naturally formed on Al surfaces has an amorphous structure [34]. When heat-treated at temperatures below about 500 °C, an alumina film grows while maintaining the amorphous structure. When heat treatment is performed at temperatures above 500 °C, crystallized γ-alumina is formed, and the film thickness increases significantly. Therefore, the oxidation heat treatments were carried out in air at 400–550 °C for a fixed duration of 10.8 ks. Auger electron spectroscopy (AES, PHI 710, ULVAC-PHI Inc., Kanagawa, Japan) was used to quantify the oxidation state of the Al powder surface. By analyzing the Auger electrons detected from a single Al powder, elemental concentration profiles were obtained in the depth direction from the Al powder surface. The oxygen concentration profile shows a high concentration at the surface due to the presence of alumina. From the surface to the interior of the particle, the oxygen concentration decreases rapidly and reaches a nearly constant value. The oxide film thickness was defined as the depth at which the oxygen concentration reached an intermediate concentration between the particle surface and interior. Under this definition, the oxide film thickness on the as-supplied Al powder was approximately 2 nm.

Table 3 shows the combinations of oxidation heat treatments and Al-X aids used in this study. For each of these combinations, the powders were weighed and mixed manually for 1.2 ks to achieve the compositions shown in Figure 1. The powder mixture was filled into a mold with an inner diameter of 10 mm and cold pressed at 25–80 MPa to produce a cylindrical compact with a relative density of approximately 75%. The setup for sintering the Al powder compacts is shown in Figure 3b. Alumina particles with a particle size of 0.3 μm were put in an alumina crucible to create a flat area for the sample to be set on the rounded-bottom crucible. The Al powder compact was placed on the alumina particles. The crucible was put in a graphite crucible set in a high-frequency induction furnace. A thermocouple was placed just above the Al powder compact to measure the temperature. The furnace was evacuated to approximately 20 Pa, followed by introducing Ar gas of approximately 0.08 MPa. Then, the Al powder compact was heated up to 630 °C at a rate of 0.33 °C·s^−1^ and sintered for 0–1800 s. The furnace power was turned off to cool the sample to ambient temperature. A representative thermal history during the sintering is shown in Figure 3c. The relative density of the sintered samples was measured using the Archimedes method. The samples were then embedded in epoxy resin and subjected to mechanical polishing with SiC polishing paper, buffing with 3 μm and 1 μm diamond slurries, and finishing polishing with colloidal silica. The microstructure of the sintered samples was observed by scanning electron microscopy (SEM, JSM-6610A, JEOL, Tokyo, Japan). Elemental distribution was analyzed by energy-dispersive X-ray spectroscopy (EDS). The constituent phases were analyzed by X-ray diffraction (XRD) measurement using Cu-Kα radiation operated at 40 kV and 40 mA. Cu-Kβ radiation was also generated but mostly cut by a Ni filter (the sample is irradiated with a slight Cu-Kβ radiation).

## 3. Results

### 3.1. Sintering Behavior and Microstructure of Samples Sintered Using As-Supplied Al Powder

**Figure 4:**
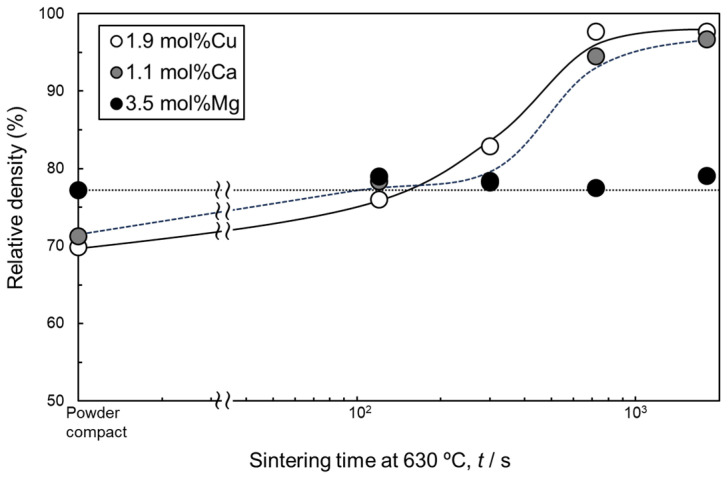
Change in relative density of samples liquid-phase-sintered by Al-Cu, Al-Ca, and Al-Mg eutectic alloy agents as a function of sintering time at 630 °C.

Figure 4 shows the change in the relative density of samples sintered at 630 °C using as-supplied Al powder compacts with Al-Cu, Al-Ca, and Al-Mg aids as a function of sintering time. Comparing the relative densities of the sintered compacts after 1800 s, the relative density of the compacts with Al-Cu and Al-Ca aids reached approximately 96–97%. In contrast, the relative density of the sintered compacts with the Al-Mg aid was approximately 80%, which is lower than that of the compacts with the other two aids. The densification of the samples with Al-Cu and Al-Ca aids was similar. In particular, densification progressed significantly between 300 and 720 s. When Al-Mg was used, the relative density remained almost constant regardless of the sintering time.

**Figure 5:**
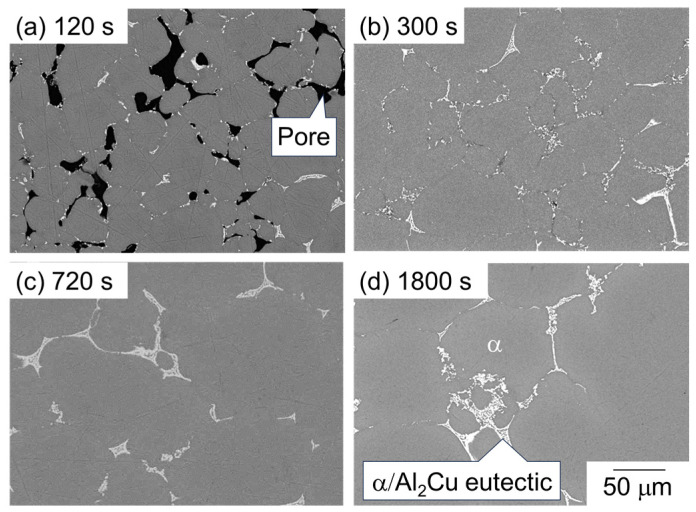
SEM images showing the microstructure of samples sintered with Al-Cu aid at 630 °C for (**a**) 120 s, (**b**) 600 s, (**c**) 720 s, and (**d**) 1800 s.

Figure 5 shows the SEM images of the microstructure of the sample sintered using as-supplied Al powder compacts with Al-Cu aid. The dark and bright areas represent pores and the θ-Al_2_Cu phase, respectively, and the intermediate area indicates the α phase. In the microstructure of the sample sintered for 1800 s, the Al_2_Cu phase or α/Al_2_Cu eutectic structure surrounding the α phase is observed (Figure 5d). Based on the equilibrium phase diagram for the Al-Cu binary system, the α/Al_2_Cu eutectic would be formed by the partitioning of Cu into the liquid phase during the cooling and the solidification of the liquid phase. That is, it can be considered that the liquid phase was present during sintering at the areas where the eutectic structure was observed. Many pores were observed at 120 s but were significantly reduced to 300 s (Figure 5a,b). A significant grain growth of α-grains was observed between 300 s and 720 s (Figure 5b,c).

**Figure 6:**
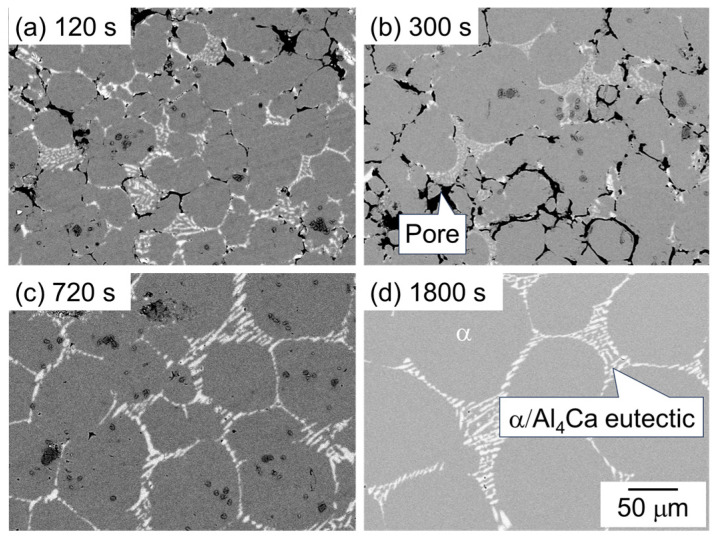
SEM images showing the microstructure of samples sintered with Al-Ca aid at 630 °C for (**a**) 120 s, (**b**) 600 s, (**c**) 720 s, and (**d**) 1800 s.

Figure 6 shows the SEM image showing the microstructure of samples sintered using as-supplied Al powder with the Al-Ca aid. The dark and bright areas represent pores and Al_4_Ca, respectively, and the intermediate area indicates the α-phase. A distinct α/Al_4_Ca eutectic structure was observed in the sample sintered for 1800 s (Figure 6d), indicating that this region was the liquid phase during sintering. While many pores were observed at 120 s and 300 s, the number of pores decreased significantly after 720 s, and the densification progressed. The α-grain growth progressed rapidly between 300 s and 1800 s.

**Figure 7:**
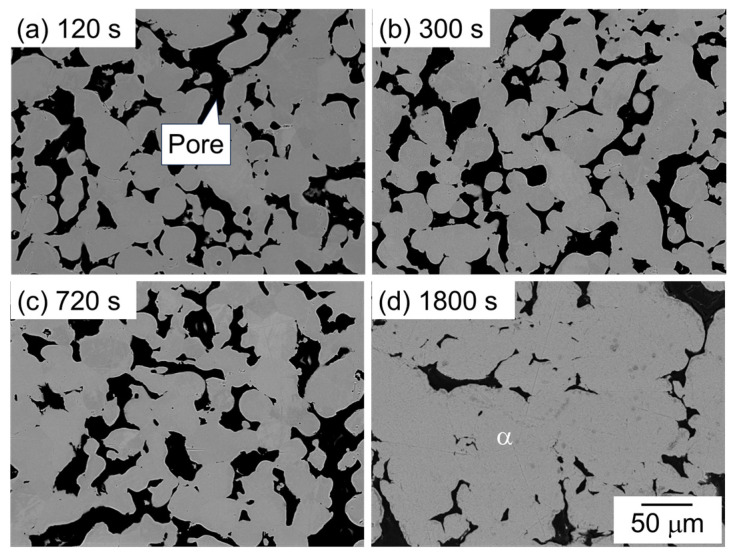
SEM images showing the microstructure of samples sintered with Al-Mg aid at 630 °C for (**a**) 120 s, (**b**) 600 s, (**c**) 720 s, and (**d**) 1800 s.

Figure 7 shows the SEM image showing the microstructure of samples sintered using the as-supplied Al powder compact with Al-Mg aid. The dark and bright areas represent pores and α phase, respectively. One major difference from the other two samples with Al-Cu and Al-Ca aids is that no clear eutectic structure was observed. In addition, many pores were observed at all sintering times, indicating that the densification hardly progressed. These results indicate that Al-Cu and Al-Ca aids promote the densification of Al powder compact, but Al-Mg aid contributes little to densification.

**Figure 8:**
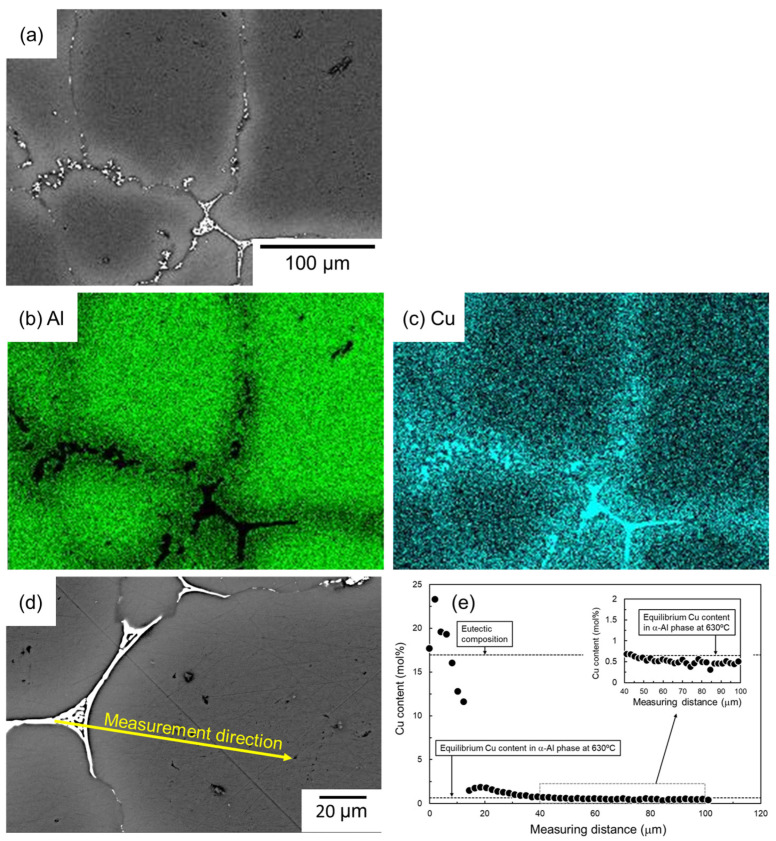
(**a**) SEM images and (**b**,**c**) corresponding EDS element maps of (**b**) Al and (**c**) Cu. (**d**) SEM image showing the EDS line analysis direction and (**e**) the corresponding Cu content.

Figure 8 shows the SEM images and the corresponding EDS elemental analysis results of the sample sintered using as-supplied Al powder compact sintered with Al-Cu aid for 1800 s. A significant Cu enrichment can be seen in the eutectic structure. Cu enrichment is not observed inside the α-grain, but Cu enrichment can be confirmed in the vicinity of the eutectic zone. The results of EDS line analysis along the line from the eutectic zone to the center of the α-grain, shown in the SEM image in Figure 8d, are shown in Figure 8e. At the eutectic zone, Cu concentrations have a large variation but are generally around 17.3 mol% (α/Al_2_Cu eutectic composition), supporting the notion that the eutectic zone is composed of α/Al_2_Cu two-phase. The Cu concentration near the center of the α-grain was lower, but a Cu concentration of approximately 0.5 mol% was quantified. This is close to the equilibrium Cu concentration of the α-phase in the α/liquid two-phase region at 630 °C. This suggests that the Cu concentration in the α-phase has almost reached equilibrium after sintering at 630 °C/1800 s. Considering that the diffusion of atoms in the liquid phase is much faster than in the solid phase, the Cu concentration in the liquid phase would have almost reached equilibrium during sintering. It is noted here that the Cu concentration in the α-phase near the eutectic zone was higher than around the center of the α-grain and reached approximately 2 mol%. The Cu enrichment corresponded to the EDS element map shown in Figure 8c.

**Figure 9:**
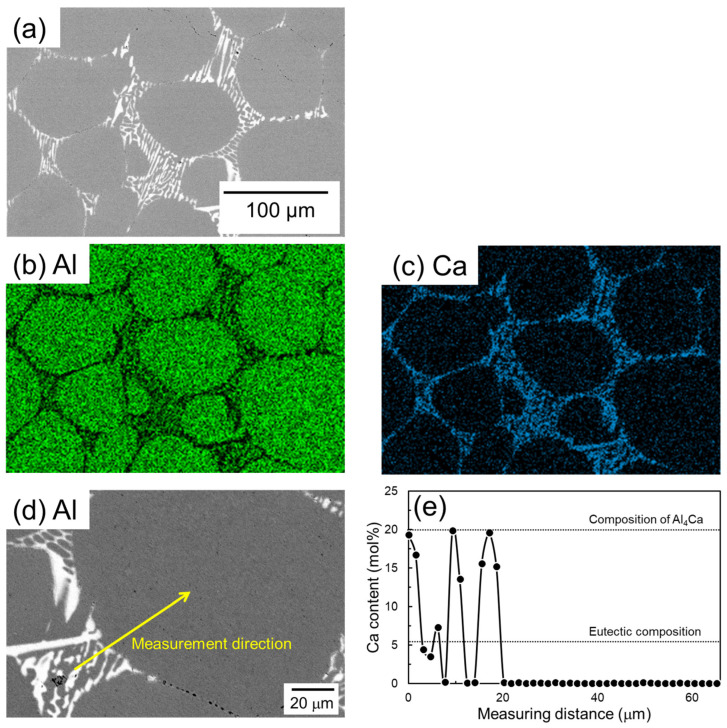
(**a**) SEM images and (**b**,**c**) corresponding EDS element maps of (**b**) Al and (**c**) Ca. (**d**) SEM image showing the EDS line analysis direction and (**e**) the corresponding Ca content.

Figure 9 shows SEM images and corresponding EDS elemental analysis results of a sample of Al powder sintered for 1800 s using Al-Ca aid. Ca is concentrated in the eutectic zone. Unlike the case with the Al-Cu aid, there is no Ca concentration in the α-grain near the eutectic zone. The EDS line composition analysis was performed from the eutectic zone to the center of the α-grain along the line shown in Figure 9d, and the results are shown in Figure 9e. In the eutectic zone, there is a significant variation in Ca concentration. The maximum Ca concentration detected was approximately 20 mol%, which corresponds to the Ca concentration in the Al_4_Ca phase. The minimum value in the variation is almost 0 mol%, corresponding to the α phase. These results support the notion that this eutectic zone is composed of an α/Al_4_Ca two-phase. The Ca concentration in the α grains was almost 0 mol%. This corresponds well to the Ca solid solubility limit of nearly 0 mol% in the α phase in the equilibrium phase diagram of the Al-Ca binary system (Figure 1b).

**Figure 10:**
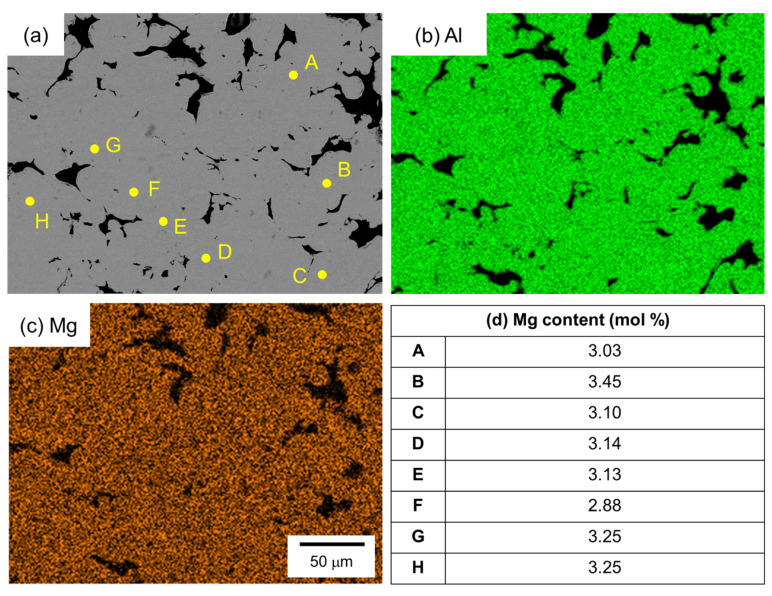
(**a**) SEM images and (**b**,**c**) corresponding EDS element maps of (**b**) Al and (**c**) Mg. (**d**) Mg content measured by EDS analyses at the points shown in (**a**).

Figure 10 shows the SEM images and the corresponding EDS elemental analysis results of the sample sintered from the as-supplied Al powder for 1800 s using Al-Mg aid. Unlike the case with Al-Cu and Al-Ca aids, no eutectic zone was observed, and no clear Mg enrichment was observed in the EDS element map. Therefore, EDS point analysis was performed at randomly selected positions in the α phase. The results are shown in Figure 10d. A Mg concentration of around 3.2 mol% was detected, although there was a slight variation. This value is close to the average Mg concentration of 3.5 mol% in this sample. Thus, Mg was uniformly distributed throughout the sample.

### 3.2. Sintering Behavior and Microstructure of Samples Sintered Using Oxidation Heat-Treated Al Powder

**Figure 11:**
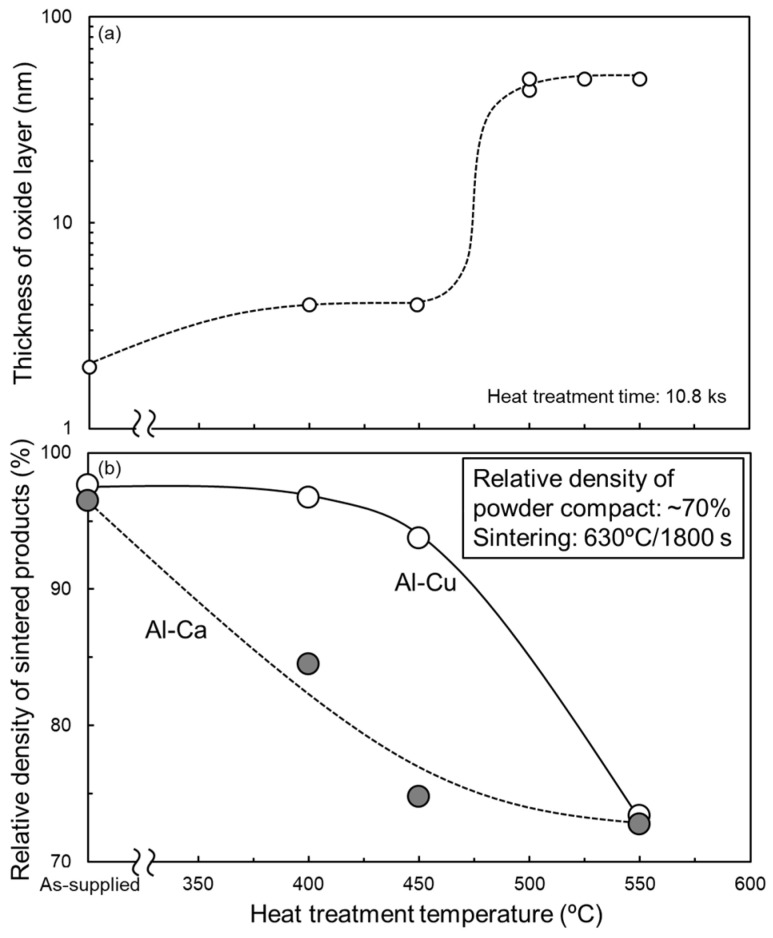
(**a**) Change in thickness of Al oxide film covered on Al powder as a function of oxidization heat treatment temperature under air atmosphere (heat treatment time: 10.8 ks). (**b**) Relationship between the relative density of samples sintered at 630 °C for 1800 s using Al powder oxidized under an air atmosphere and oxidization heat treatment temperature.

From Section 3.1, Al-Cu and Al-Ca aids effectively promote the sintering of as-supplied Al powder compacts. To clarify the feasibility of these aids for sintering oxidized Al powders, the oxidation heat-treated Al powder compacts were sintered using these aids. Figure 11a shows the relationship between the thickness of the oxide film on the surface of the oxidation heat-treated Al powder and the heat treatment temperature. When the heat treatment temperatures were 400 °C and 450 °C, the thickness of the oxide film was approximately 4 nm, roughly twice as thick as that of the as-supplied Al powder. When heat-treated at 500 °C–550 °C, the oxide film thickness was approximately 50 nm, which is approximately 25 times thicker than that of the as-supplied Al powder. These trends are consistent with previous studies [34] and suggest that the growth of the oxide film at 400 °C and 450 °C would be due to the amorphous Al_2_O_3_ and that the rapid growth of the oxide film at 500–550 °C would be due to the formation of γ-Al_2_O_3_. The relative densities of the samples sintered at 630 °C/1800 s using the oxidized Al powders with Al-Cu and Al-Ca aids are shown in Figure 11b. When Al powder was oxidized at 550 °C (the oxide film thickness was increased to approximately 50 nm), the relative density significantly degraded to approximately 73% regardless of whether Al-Cu or Al-Ca aids were used. This relative density was almost the same as the relative density of the powder compacts before sintering, indicating that densification hardly progressed. Thus, it was difficult to sinter Al powder with an oxide film grown to about 50 nm (γ-Al_2_O_3_) through the liquid phase sintering process using eutectic alloy powders. When the Al powders were oxidized at 400 °C or 450 °C (the oxide film thickness was approximately 4 nm), the relative density of the sintered samples was highly dependent on the sintering aids. When sintered using Al-Cu aids, the relative density maintained relatively high values of approximately 94–96%. The Al powder compacts with an oxide film thickness of approximately 4 nm can be densified using the Al-Cu aid. In contrast, the relative density of the samples sintered using the Al-Ca aid was significantly reduced to about 85% and 75%, even when the Al powder was oxidized at 400 °C and 450 °C, respectively. The liquid phase sintering with the Al-Ca aid was very sensitive to the oxidation state of the Al powders.

**Figure 12:**
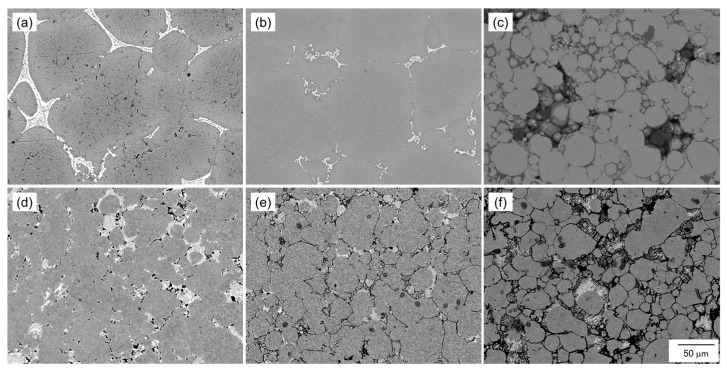
SEM images showing the microstructure of the samples sintered using aluminum powder oxidized at (**a**,**d**) 400 °C, (**b**,**e**) 450 °C, and (**c**,**f**) 550 °C for 10.8 ks. (**a**–**c**) Al-Cu and (**d**–**f**) Al-Ca alloy powders.

Figure 12 shows SEM images showing the microstructure of the samples sintered at 630 °C/1800 s using the oxidized Al powders with Al-Cu and Al-Ca aids. When the Al powder was oxidized at 400 °C or 450 °C and sintered using Al-Cu aids, the α-grains surrounded by the eutectic zone showed significant grain growth (Figure 12a,b). However, when the Al powder oxidized at 550 °C was sintered using the Al-Cu aid, the shape of the raw Al powder was almost maintained, and the grain growth hardly occurred (Figure 12c). When the Al powder oxidized at 400 °C was sintered using the Al-Ca aid, regions with the size of the raw powder surrounded by the eutectic zone were observed, indicating that grain growth was suppressed compared to when sintered using the Al-Cu aid (Figure 12d). When the Al powder oxidized at 450 °C was sintered using the Al-Ca aid, clear grain boundaries were observed, and the α grain size was approximately 50 μm, indicating that grain growth was greatly suppressed (Figure 12e). Furthermore, when the Al powder oxidized at 550 °C was sintered using the Al-Ca aid, Al powder maintained the shape of the raw powder, and the grain growth hardly occurred (Figure 12f), similar to when sintered using the Al-Cu aid.

**Figure 13:**
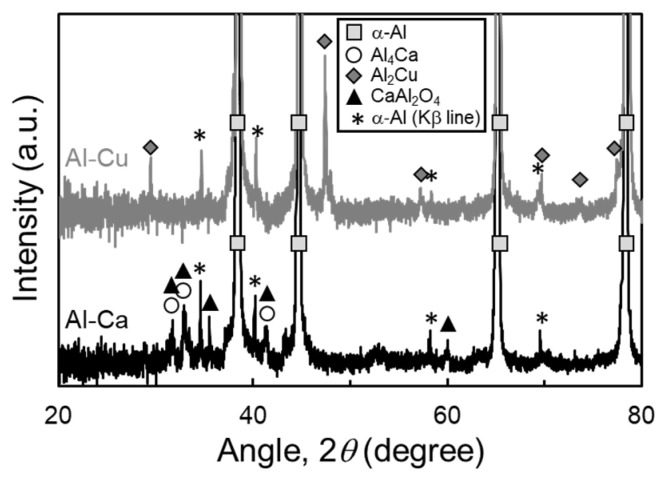
X-ray diffraction (XRD) profile of samples sintered at 630 °C for 1800 s using Al powder oxidized at 450 °C for 10.8 ks together with Al-Cu and Al-Ca aids.

Figure 13 shows the XRD profile of samples sintered at 630 °C for 1800 s using Al powder oxidized at 450 °C for 10.8 ks together with Al-Cu and Al-Ca aids. The peaks of the α and Al_2_Cu phases were detected in the sample sintered using the Al-Cu aid. The peaks of α-Al and Al_4_Ca phases were detected in the sample sintered using the Al-Ca aid, but a few peaks did not correspond to these phases. The peaks corresponded to the CaAl_2_O_4_ phase, suggesting that Ca in the Al-Ca melt may react with the Al_2_O_3_ phase to form the CaAl_2_O_4_ phase. It has been reported that the CaAl_2_O_4_ phase was formed at the interface between Al-Ca melt and Al_2_O_3_ particles in the Al-Ca/Al_2_O_3_ composites fabricated through gas pressure-assisted melt infiltration at 950 °C [30]. A previous study supports the formation of the CaAl_2_O_4_ phase, although the temperature of this previous study was much higher than that of this study. As shown in Figure 1b, the equilibrium Ca concentration of the Al-Ca liquid phase is approximately 3 mol% (=4.3 mass%). The CaAl_2_O_4_ phase was formed as a continuous layer when Al-4 mass%Ca came into contact with Al_2_O_3_. Therefore, the CaAl_2_O_4_ phase might be formed as a continuous layer by the reaction between Al-Ca melt and Al_2_O_3_ film on Al powder, inhibiting the densification and grain growth. To demonstrate the hypothesis above, minute transmission electron microscopy (TEM) observations are required in future studies.

These results show that densification and grain growth were very sensitive to the oxidation state of the Al powder surface in liquid phase sintering using the Al-Ca aid. When the Al powder was sintered using the Al-Ca aid, the oxidation of the Al powder surface needed to be strictly suppressed. In contrast, when the Al-Cu aid was used, densification and grain growth progressed even when the Al powder was oxidized at 400 °C or 450 °C (oxide film thickness of approximately 4 nm), although sintering of Al powder with an oxide film of approximately 50 nm (related to the formation of γ-Al_2_O_3_) was difficult. This suggests that the Al powders produced by various manufacturing routes (e.g., N_2_, air, and water atomizations [15,35]) may also be sintered using Al-Cu aid, although the oxidation state of the Al powders needs to be investigated in detail. Thus, it can be concluded that the Al-Cu aid is the most appropriate among the eutectic alloy aids investigated in this study.

## 4. Discussion

Among the aids investigated in this study, only the Al-Mg aid did not promote the densification of as-supplied Al powder compacts (Figure 4 and Figure 7). The reasons are discussed here. In a previous study of the liquid phase sintering process using Mg elemental powder, the addition of a small amount of Mg (below 1 mass%) promoted densification through the reaction with the oxide film, while the addition of a large amount of Mg (over 1 mass%) conversely caused the swelling of the specimens [36]. It is generally known that swelling occurs in the early stage of liquid phase sintering when atomic diffusion from the liquid phase to the solid phase is dominant due to a large solubility limit of the additive elements in the solid phase [33]. Mg has the largest solid solubility limit in the α phase among the elements investigated in this study (Figure 1a–c) and is therefore likely to cause swelling. However, the conditions for liquid phase sintering were carefully tailored based on the equilibrium phase diagram and set so that the liquid phase exists continuously during sintering and the liquid phase fraction increases from the stage of aid melting to the equilibrium (Figure 1d–f). The increase in the liquid phase fraction means that atomic diffusion from the solid phase to the liquid phase is more pronounced than atomic diffusion from the liquid phase to the solid phase, which is less likely to cause swelling. The sample sintered using Cu-containing aid (the second highest solubility limit in this study) reached a high relative density of 97%, which was as high as the sample sintered using the Ca-containing aid (solubility limit of nearly 0 mol%). The reason why the densification of the sample sintered using the Al-Mg aid did not progress would not be the swelling during sintering due to the large solubility limit.

**Figure 14:**
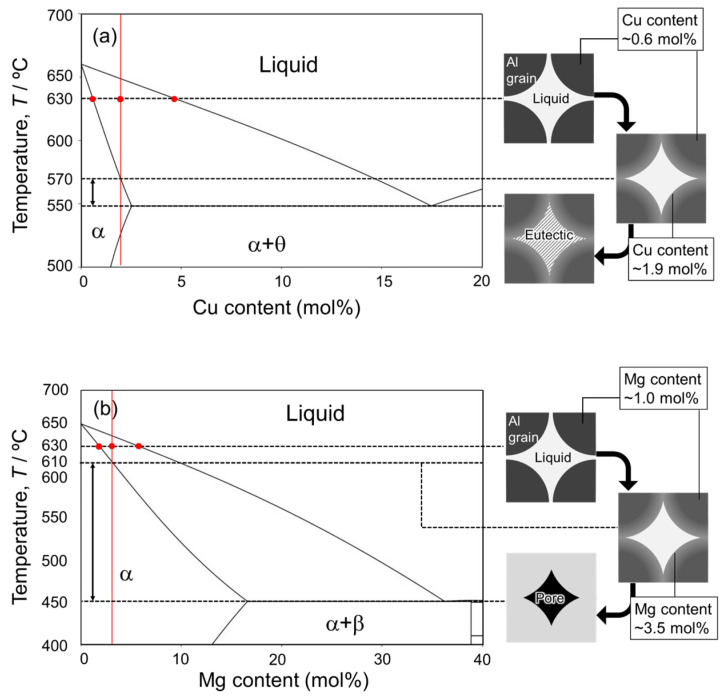
Equilibrium phase diagrams and schematic illustration during the cooling process after sintering of Al powder assisted by (**a**) Al-Cu and (**b**) Al-Mg alloy agents.

As shown in Figure 5, Figure 6, Figure 8 and Figure 9, Cu- or Ca-enriched eutectic zones were present when the Al-Cu and Al-Ca aids were used. The liquid phase would be present during sintering, where the eutectic zone was observed. In contrast, as shown in Figure 7 and Figure 10, no eutectic zone was observed when the Al-Mg aid was used, and Mg was almost uniformly distributed. However, the sintering was performed under conditions where the liquid phase continuously existed at 630 °C. The liquid phase also existed at 630 °C when the Al-Mg aid was used. Therefore, diffusion during the cooling process may be the reason why no Mg-enriched eutectic zone was present in the sample sintered using the Al-Mg aid. Figure 14 shows schematic illustrations of the phenomena that may occur during the cooling process. In this study, the cooling rate was relatively slow and approximately 10^−1^ °C·s^−1^ as shown in Figure 3c. The slow cooling rate indicates that the sintered microstructure at 630 °C was not necessarily preserved. For example, Cu concentration in the α-grains near the eutectic zone was higher than the equilibrium Cu concentration at 630 °C (Figure 8e) when the Al-Cu aid was used. At 630 °C/1800 s, the Cu concentration in α phase reached 0.6 mol% (Figure 8e and Figure 14a). During the cooling process, the solid phase fraction increases, and the liquid phase fraction decreases, which causes the solid phase to grow toward the liquid phase. Since local equilibrium would be established at the solid–liquid interface due to the relatively slow cooling rate, the Cu concentration on the solid phase at the solid–liquid interface at each temperature corresponded to the solidus line in the phase diagram. As a result, Cu of a higher concentration than the equilibrium Cu concentration at 630 °C was partitioned into the solid phase, while Cu was highly concentrated in the liquid phase along the liquidus line. The composition of Al-1.9 mol% intersects the solidus line at 570 °C. That is, at temperatures below 570 °C, the equilibrium state is the α single phase without the liquid phase. However, since 570 °C is close to the eutectic temperature of 548 °C, the Cu-enriched liquid phase decomposed into the Al/Al_2_Cu two phases by eutectic reaction at 548 °C prior to the homogenization of the α single phase. Atomic diffusion would be suppressed, and no major microstructural changes would occur since the liquid phase would have disappeared at this stage. As a result, the Al/Al_2_Cu eutectic structure at the α-grain boundary and a high Cu concentration of approximately 2 mol% within the α-grain in the vicinity of the Al/Al_2_Cu eutectic structure were observed.

It is assumed that the discussion is equally applicable to the sample sintered using Al-Mg aid; the Mg concentration in the α phase would reach the equilibrium at 630 °C and increase at the solid–liquid interface along the solidus line during the cooling. The composition of Al-3.5 mol%Mg intersects the solidus line at a temperature of approximately 610 °C (Figure 14b). Below 610 °C, the α-single phase is the equilibrium state. The eutectic temperature of the Al-Mg system is 450 °C, which is much lower than 610 °C. This large difference between solidus and eutectic temperatures presumably causes the homogenization of the α single phase during the cooling, resulting in the absence of the eutectic or Mg-enriched zone in the sintered sample. To quantitatively verify the homogenization during the cooling, the diffusion distances of Cu and Mg were calculated. The diffusion coefficient (*D*) is expressed as the following Arrhenius equation.

(1) $$D=D_{0}\exp(-\frac{Q}{RT})$$

Here, *D*_0_ is the frequency factor, *Q* is the activation energy, *R* is the gas constant, and *T* is the absolute temperature. According to the literature [37], *D*_0_ = 6.2 × 10^−6^ m^2^·s^−1^ and *Q* = 115 kJ·mol^−1^ for the diffusion of Mg in Al, and *D*_0_ = 6.5 × 10^−5^ m^2^·s^−1^ and *Q* = 136 kJ·mol^−1^ for the diffusion of Cu in Al. The one-dimensional diffusion distance for isothermal processes is calculated by the following equation.

(2) $$L=\sqrt{2Dt}$$

Here, *t* is the holding duration in an isothermal process. Although the diffusion during the cooling is not an isothermal process, it can be considered as a repetition of isothermal holding for a short duration and instantaneous temperature lowering. Here, the cooling process from the temperature at which the α-phase single phase reaches equilibrium (570 °C for Al-Cu, 610 °C for Al-Mg) to the eutectic temperature (548 °C for Al-Cu, 450 °C for Al-Mg) was considered using the temperature history shown in Figure 3c. The diffusion distances were approximately 4 μm for the sample sintered using the Al-Cu aid and approximately 16 μm for the sample sintered using the Al-Mg aid. Considering the diffusion from the liquid phase at the α-grain boundary to the α-grain interior, the diffusion distance required for the homogenization should be the radius of the α-grain. Since the Al powder size used in this study was <45 μm, the diffusion distance of 16 μm made it possible to diffuse Mg to the center of the α-grain, whereas the diffusion distance of 4 μm made it difficult to diffuse Cu to the center of the α-grain. Thus, the homogenization of the α single phase during the cooling would be the reason for the absence of eutectic or Mg-enriched zones in the sample sintered using the Al-Mg aid. As shown in the schematic illustration in Figure 14, the liquid phase disappeared from α grain boundaries. However, the growth of α solid phase would not compensate for the lost volume of the liquid phase with a higher volume than the solid phase (solidification shrinkage of Al was approximately 6.5% [37]). Therefore, pores would form where the liquid phase existed. However, in situ observations of the sintering and subsequent cooling processes are required to verify the pore formation mechanism. Recently, in situ synchrotron X-ray computed tomography (CT) has been applied to observe the sintering sequence of alumina powders [38]. If the spatial and time resolutions of the in-situ observations are sufficient, the pore formation mechanism of samples sintered using Al-Mg aid can be clarified by observing distributions of pores and liquid phase during sintering and after cooling.

The discussion also suggests that densification may have occurred during sintering when the Al-Mg aid was used, but that pores may have formed during the subsequent cooling process. There are possibilities that Al powder compacts can be densified using the Al-Mg aid by solidifying the liquid phase through the eutectic reaction before the homogenization of the α single phase. One is to increase the cooling rate. The second is to lower the temperature at which the composition and solidus line intersect by increasing the Mg concentration and lowering the sintering conditions. Both approaches would solidify the liquid phase as the eutectic before the homogenization of the α single phase and may fabricate dense samples. The effects of cooling rate, Mg concentration, and sintering temperature on the relative density and microstructure of the sample sintered using the Al-Mg aid need to be investigated.

## 5. Conclusions

In this study, liquid phase sintering of Al powder compacts (relative density: about 75%) using Al-X (X=Cu, Ca, Mg) eutectic alloy powders as sintering aids was performed, and the relative density and microstructure of the sintered compacts were evaluated. The oxidation state of the Al powder surface was varied by oxidization heat treatment under atmospheric conditions, and the effect of oxide film thickness on the liquid phase sintering behavior using each aid was investigated. The main findings are listed as follows:

- Sintering using as-supplied Al powder at 630 °C/1800 s resulted in a high relative density of 96–97% when the Al-Cu and Al-Ca aids were used. However, when the Al-Mg aid was used, the relative density was limited to approximately 80%.
- The eutectic structures (assuming the liquid phase during the sintering) were observed when the Al-Cu and Al-Ca aids were used, while no eutectic structure was observed, and Mg was uniformly distributed when Al-Mg aid was used.
- The homogenization of the α single phase during the cooling process would be the reason why Mg is uniformly distributed when the Al-Mg aid is used. The wide temperature range from the solidus temperature to the eutectic temperature would provide sufficient time to diffuse Mg to the center of the α-grain, causing the formation of pores at the areas where the liquid phase existed.
- When Al powder was heat-treated at 550 °C to thicken the oxide film to approximately 50 nm and subsequently sintered at 630 °C/1800 s using Al-Cu or Al-Ca aids, densification hardly progressed. Al powder with a significantly grown oxide film on the surface is difficult to sinter using eutectic alloy powders.
- When Al powder was heat-treated at 400–450 °C to thicken the oxide film to approximately 4 nm and subsequently sintered at 630 °C/1800 s, the densification and grain growth occurred using the Al-Cu aid, but the relative density and grain growth of the sample sintered using the Al-Ca aid were significantly suppressed. This would be due to the oxides produced by the reaction of Ca and Al_2_O_3_, which would inhibit the progress of sintering and grain growth.
- It can be concluded that the Al-Cu aid, which is relatively insensitive to the oxidation state of the Al powder surface and can achieve a high relative density, is the most appropriate liquid phase sintering aid among those investigated in this study.

As concluding remarks, a suitable Al-Cu aid for the liquid phase sintering of Al powder was revealed in this study. To clarify the reason why Al-Cu aid is more suitable than Al-Mg and Al-Ca aids in more depth, in situ observations using synchrotron X-ray radiation and TEM observations need to be performed. Considering this and previous studies [37], Al-Si, Al-Cu, and their combined Al-Cu-Si aids can be applied to the liquid phase sintering of Al powder. Liquid phase sintering aid containing Cu will especially contribute to a good balance of high strength and conductivity [39] due to the age hardening of sintered products, but it will have a detrimental effect on corrosion resistance [40]. In order to further select appropriate aids to each application, it is necessary to evaluate the mechanical properties, corrosion resistance, and thermal or electrical conductivities of samples sintered using these aids. Using appropriate aids to BJT and MEX after clarifying these end-use properties, complex-shaped parts, including automobile parts and heat exchangers, will be manufactured. However, the shrinkage of the product from the green body is inevitable for the sintering process. Therefore, the dimensional accuracy of the liquid phase sintering process using Al-Cu aid needs to be investigated.

## Figures and Tables

**Table 1:** The eutectic composition, eutectic temperature, and maximum solubility limit of each alloy element in the α phase at the eutectic temperature of Al-X (X=Mg, Ca, and Cu) eutectic alloys.

	Al-Mg	Al-Ca	Al-Cu
Eutectic composition (mol%)	37	5.5	17.3
Eutectic temperature (°C)	450	613	548
Maximum solubility limit (mol%)	16.5	0	2.5

**Table 2:** Elemental compositions measured by induction coupled plasma (ICP) analysis (Mg, Ca, Cu, Si, and Fe) and the infrared absorption method (O). Hyphens (-) indicate that the elements were not measured.

Alloy	Composition (mass%)					
	Cu	Mg	Ca	Si	Fe	O
Al-17.3 mol%Cu (Al-33 mass%Cu)	32.77	-	-	<0.01	<0.01	0.082
Al-37 mol%Mg (Al-35 mass%Mg)	-	34.85	-	<0.01	<0.01	0.20
Al-5.5 mol%Ca (Al-8 mass%Ca)	-	-	7.85	<0.01	<0.01	-

**Table 3:** Combinations of heat treatment conditions of Al powder and kinds of eutectic alloy aid experimentally carried out in this study. The circle (●) and hyphen (-) indicate that the conditions were carried out and not carried out, respectively.

Condition of Al Powder	Eutectic Alloy Agent		
	Al-Cu	Al-Ca	Al-Mg
As-supplied	●	●	●
400 °C heat-treated	●	●	-
450 °C heat-treated	●	●	-
550 °C heat-treated	●	●	-

## Data Availability

The original contributions presented in this study are included in the article. Further inquiries can be directed to the corresponding author.
